# The Role of the Clathrin Adaptor AP-1: Polarized Sorting and Beyond

**DOI:** 10.3390/membranes4040747

**Published:** 2014-11-07

**Authors:** Fubito Nakatsu, Koji Hase, Hiroshi Ohno

**Affiliations:** 1 Department of Cell Biology, Yale University School of Medicine, 295 Congress Avenue, BCMM237, New Haven, CT 06510, USA; 2 Department of Biochemistry, Faculty of Pharmacy, Keio University, Tokyo 105-8512, Japan; E-Mail: hase-kj@pha.keio.ac.jp; 3 RIKEN Center for Integrative Medical Sciences (IMS), Kanagawa 230-0045, Japan; E-Mail: ohno@rcai.riken.jp

**Keywords:** AP complex, μ1B, clathrin adaptor, vesicular transport, sorting signal, polarized sorting, epithelial cell, inflammation, cancer

## Abstract

The selective transport of proteins or lipids by vesicular transport is a fundamental process supporting cellular physiology. The budding process involves cargo sorting and vesicle formation at the donor membrane and constitutes an important process in vesicular transport. This process is particularly important for the polarized sorting in epithelial cells, in which the cargo molecules need to be selectively sorted and transported to two distinct destinations, the apical or basolateral plasma membrane. Adaptor protein (AP)-1, a member of the AP complex family, which includes the ubiquitously expressed AP-1A and the epithelium-specific AP-1B, regulates polarized sorting at the trans-Golgi network and/or at the recycling endosomes. A growing body of evidence, especially from studies using model organisms and animals, demonstrates that the AP-1-mediated polarized sorting supports the development and physiology of multi-cellular units as functional organs and tissues (e.g., cell fate determination, inflammation and gut immune homeostasis). Furthermore, a possible involvement of AP-1B in the pathogenesis of human diseases, such as Crohn’s disease and cancer, is now becoming evident. These data highlight the significant contribution of AP-1 complexes to the physiology of multicellular organisms, as master regulators of polarized sorting in epithelial cells.

## 1. Introduction

Intracellular vesicular transport is a fundamental membrane trafficking process that governs the transport of proteins and lipids via small membranous carrier vesicles [1,2]. Various organelles send and receive these carrier vesicles to transport or exchange materials among them, and this constitutes a housekeeping function that supports cellular and physiological activities. At the same time, the membrane composition of each organelle must be kept separated and not be intermixed so that each organelle maintains its own property and fulfills a unique function. Thus, the intracellular vesicular transport system is tightly controlled to achieve a highly sophisticated, active and seamless membrane transport by keeping the membrane “identity” of each organelle or constituting membrane domain.

Cells evolutionarily develop their own intracellular vesicular transport networks. Such networks differ in their own complexity and diversity, depending on the organism or cell type. Multicellular organisms, including higher mammals, harbor many different types of cells, each with a unique set of functions. Cells often possess a unique system for vesicular transport. For example, the epithelial cells are polarized cells containing two biochemically distinct plasma membrane domains, *viz*. an apical and a basolateral plasma membrane domain [3]. These plasma membrane domains are physically separated by junction structures, including adherens and tight junctions [4]. These junctional complexes prevent the spontaneous diffusion of domain-specific membrane proteins and lipids. Such an asymmetric distribution of membrane components is essential for the function of the epithelium. However, in order to establish and maintain this polarization, proteins and lipids need to be transported by distinct ways from the non-polarized cells [5]. Epithelial cells achieve this task in part by taking advantage of the polarized vesicular transport, in which the apical or basolateral transport is operated by machineries specific to each pathway. Basolateral transport is mediated by cytosolic adaptors that recognize the sorting determinants within cargo molecules, whereas the less characterized apical transport is known to be mediated through the transmembrane or extracellular region of cargo proteins themselves [5,6,7,8,9].

Adaptor protein (AP)-1, a member of the AP complex family, is a well-known machinery involved in basolateral transport, which is one of the major regulators of vesicular transport and protein sorting in eukaryotes. In this article, we have reviewed some of the recent studies highlighting the role of AP-1 and have discussed the mode of regulation of polarized sorting and vesicular transport. In particular, we have discussed the physiological contributions of the above processes in the context of multicellular organisms, including humans.

## 2. The AP Complex, an Evolutionarily-Conserved Clathrin Adaptor

**Figure 1:**
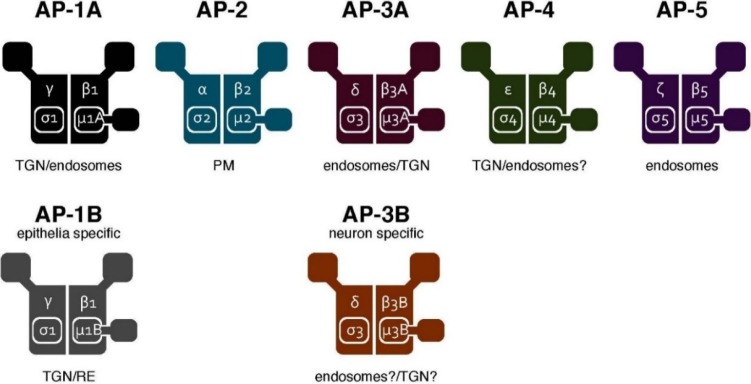
The AP complexes. Schematic representation of the AP complex family. Seven AP complexes have been identified to date. They share the overall composition of the constituting subunits: two large subunits, one medium subunit and one small subunit. The five complexes depicted at the top (AP-1A, AP-2, AP-3A, AP-4 and AP-5) are widely expressed, whereas the two complexes shown at the bottom (AP-1B and AP-3B) are expressed in a tissue-specific manner. They localize to distinct, yet partially overlapping, organelles. The organelle membrane at which each complex is localized (for controlling protein sorting) is shown below the complex. TGN, trans-Golgi network.

**Figure 2:**
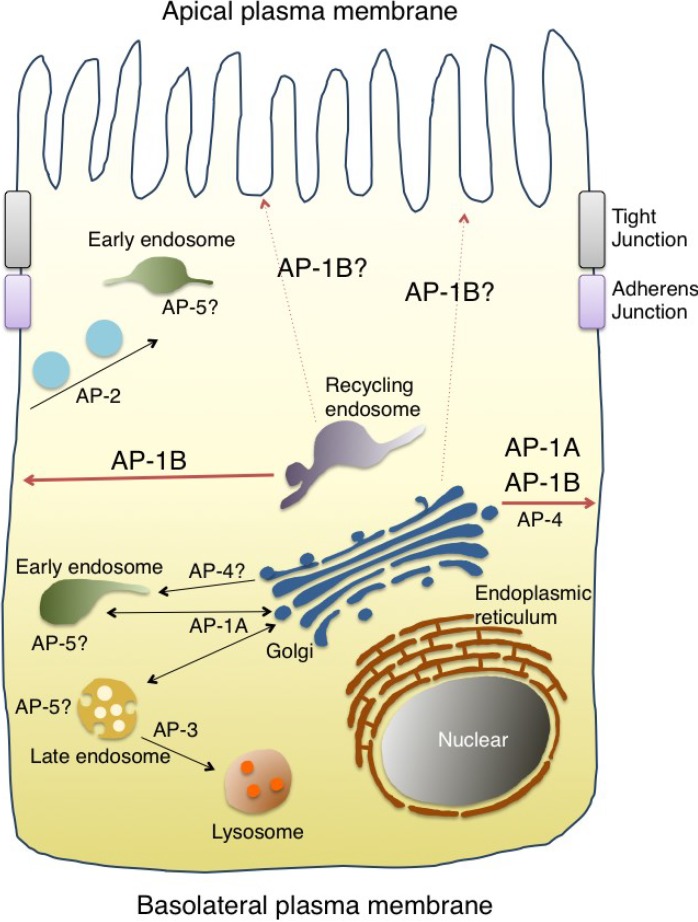
The post-Golgi network controlled by the AP complexes. The AP complexes are widely distributed throughout the post-Golgi network. AP-1A is localized at the TGN and endosomes and regulates the TGN-endosomal and the TGN-basolateral plasma membrane pathways. AP-2 is recruited to the plasma membrane for regulating endocytosis. AP-3A regulates the endosomal and the lysosomal trafficking pathways. AP-3B is involved in the biogenesis of specialized organelles, such as synaptic vesicles and dense core vesicles. AP-4 is involved in the TGN-endosomal and the TGN-basolateral plasma membrane pathways. A recently-identified AP-5 has been shown to localize at the endo-lysosomes. The epithelium-specific AP-1B and the ubiquitously-expressed AP-1A localize at the TGN and/orat the recycling endosome and control the polarized sorting to the basolateral plasma membrane. Defects in the localization of the apical proteins have been reported in a loss-of-function mutant of AP-1B, suggesting that AP-1B could be indirectly involved in the apical protein sorting. See the text for details.

The AP complex was originally identified from morphological studies (and subsequently, via biochemical characterization) as a component of the clathrin-coated vesicles in the mammalian brain [10,11,12,13,14,15,16,17]. The AP complex was subsequently found to be evolutionarily conserved in yeast [18]. It forms a heterotetrameric complex comprising two large subunits (α, β, γ, δ, ε or ζ), one medium subunit (μ1–μ5) and one small subunit (σ1–σ5), respectively. A total of seven AP complexes (including two tissue-specific types) have been identified in mammals so far [19,20] (Figure 1). They share the overall composition of a heterotetramer. However, they regulate distinct, but partially overlapping, pathways. The trans-Golgi network (TGN)-endosome pathway is regulated by AP-1 and AP-4, endocytosis by AP-2 and the endosome-lysosome pathway by AP-3 and AP-5, respectively (reviewed in [19,20,21,22]) (Figure 2).

## 3. The AP Complex Regulates Cargo Sorting and Vesicle Formation

In general, vesicular transport is a sequential event. Vesicle budding from the donor membrane is followed by transport and fusion to the acceptor membrane of the transport vesicle [23,24]. The site of action of the AP complex is the donor membrane, where the cargo molecules are loaded into the budding vesicles [25]. Once the AP complex is recruited to the donor membrane, the μ subunit or the γ/α-σ dimer recognizes and binds to the sorting signals (e.g., the tyrosine-based or di-leucine-based signals) encoded in the cargo proteins [26,27]. This essential sorting process enables the selective loading of cargo proteins into the nascent transport vesicles. Simultaneously, the AP complex also recruits clathrin (and/or some of the other accessory proteins), which propels vesicle formation via self-assembly. This eventually leads to the formation of clathrin-coated pits/vesicles. Thus, the AP complex regulates cargo sorting and vesicle formation at the budding step of vesicular transport.

The AP complexes that are widely distributed in the late secretory and endocytic pathways control the sorting and vesicular transport in the post-Golgi transport network [19] (Figure 2). Highlighting the regulatory roles of all of the AP complexes is beyond the scope of this review. Thus, we will hereafter focus our attention on AP-1 and its role in polarized sorting. Note that we have distinguished between the AP-1A and AP-1 complexes in this article. AP-1A represents the AP-1 complex harboring μ1A subunit in organisms expressing the epithelium-specific μ1B subunit while AP-1 represents the AP-1 complex in other organisms that lack such a μ1B subunit.

## 4. Discovery of the AP-1B Complex

Several studies have already suggested that basolateral sorting in the epithelial cells occurs at the TGN in a sorting signal-dependent manner, as opposed to the notion that it occurs independently [28]. Nevertheless, the molecular mechanisms underlying the polarized basolateral sorting were unknown. This problem was ultimately solved by the identification of an AP complex specifically expressed in epithelial cells.

Ohno *et al.* identified a novel μ subunit using bioinformatics analysis. Because this novel μ subunit is highly similar to the already known μ1 subunit, the newly identified μ subunit was named μ1B, and the existing μ1 was renamed as μ1A. Unlike the ubiquitously expressed μ1A, μ1B is specifically expressed in the epithelial cells from tissues including kidney, intestine, salivary gland and lung [29]. However, it is now evident that μ1B is not expressed in some epithelial cells, such as kidney proximal tubule cells [30], retinal pigment epithelial cells [31] and mouse and human hepatic cells. Interestingly, the zebrafish liver does express μ1B (see Section 7.3 for more details). Similar to the other μ subunits of the AP complexes, μ1B is also able to recognize and directly bind to a subset of tyrosine-based motifs (corresponding to a sorting signal), thereby raising an interesting possibility of the μ1B subunit mediating the polarized sorting in epithelial cells [29]. This idea was subsequently validated via cell biological characterization.

## 5. AP-1B Regulates Polarized Sorting in Epithelial Cells

Folsch *et al.* found that μ1B is incorporated into a complex containing the γ, β1 and σ1 subunits, thereby forming a new AP-1B complex in the epithelial cells. Thus, there are two AP-1 complexes in epithelial cells, *viz*. AP-1A (which has μ1A) and AP-1B (which has μ1B). More importantly, the exogenous expression of μ1B in the μ1B-negative LLC-PK1 cells (epithelial cells derived from the proximal tubules of porcine kidney) restored the basolateral localization of certain membrane proteins, including the low density lipoprotein receptor (LDLR) and the transferrin receptor (TfR) [32]. This serves as direct evidence for the regulation of basolateral sorting by AP-1B in cultured cells and a big step forward in addressing the long-term question regarding the molecular mechanisms governing basolateral sorting in epithelial cells. A loss of function of μ1B, via an siRNA-mediated knockdown in MDCK cells (a complimentary approach to the study described above [32]), confirmed the role of AP-1B in polarized sorting in cultured cells [33].

## 6. Possible Involvement of AP-1A in Polarized Sorting

Although it is now established that AP-1B regulates polarized sorting in epithelial cells, AP-1A, the ubiquitous form of AP-1, has recently been shown to be involved in polarized sorting, as well. Gravotta *et al.* found that AP-1A regulates the basolateral transport of proteins directly from the TGN, whereas AP-1B controls it from the recycling endosomes, as previously reported [33,34,35,36,37]. These observations are supported by the predominant localization of AP-1A and AP-1B (examined by the transient expression of µ1A-HA or µ1B-HA) at the TGN and at the recycling endosomes, respectively [35,38]. These results led the authors to conclude that AP-1A and AP-1B control the basolateral sorting at different organelles [38,39]. An alternative model has subsequently been published by Bonifacino and colleagues in 2013. Guo *et al.* revisited the localization of µ1A-HA and µ1B-HA with an improved approach (e.g., using a spacer between the µ subunits and the HA tag or the fluorescent protein tag) and found that AP-1A and AP-1B largely colocalize to the same extent at the TGN and at the recycling endosomes. Then how do AP-1A and AP-1B play different roles? It is found that µ1B preferentially binds a subset of sorting signals that are destined for the basolateral membrane, thereby indicating that signal recognition by the µ1 subunits, rather than the differential localization, determines their sorting function. Therefore, the authors concluded that having the µ1B expression expands the repertoire of basolateral sorting signals recognized in epithelial cells [40]. Although the proposed mechanisms for the distinct regulation of basolateral sorting by AP-1A and AP-1B (recognition of a distinct subset of sorting signals *vs.* distinct localization) are different, these studies strongly suggest that AP-1A is also capable of mediating basolateral transport in epithelial cells.

## 7. The Role of AP-1B in Tissues and Organisms

Until now, a large amount of work on AP-1B, as well as AP-1A, including the regulatory mechanisms of the polarized sorting in epithelial cells, has been done using cell culture systems and *in vitro* analysis, and some of these have already been discussed above. Considering the nature of epithelial cells, such methodologies may not be adequate to study the physiological role of AP-1, especially its role in the development and homeostasis of tissues or organs. Therefore, we will feature studies of animal models or diseases possibly associated with the functional deficits of AP-1. This may unleash certain novel aspects of its function. For more details about the cellular and molecular functions of AP-1, we would also request our readers to refer to some of the insightful reviews published elsewhere [9,41,42].

### 7.1. Nematode (Caenorhabditis elegans)

*Caenorhabditis elegans* has a set of subunits for AP-1; apg-1 for the γ subunit, apb-1 for the β1 subunit, aps-1 for the σ1 subunit and apm-1 and unc101 for the μ1 subunits, respectively. A knockdown of aps-1, apb-1 or apg-1, or a simultaneous knockdown of both of the μ1 subunits (apm-1 and unc-101) in *C. elegans*, a condition expected to inactivate the entire AP-1 complex (due to the degradation of other subunits destabilized in the absence of a subunit [43,44,45,46,47,48]), leads to growth arrest at the embryonic stage, thus suggesting an essential role of AP-1 in nematode development [49,50,51]. However, loss-of-function studies of AP-1 based on a single knockdown of each of the μ1 subunits, studies on the *unc-101* genetic mutants and reports highlighting the *in vivo* knockdown of larva or young adults, all suggest that AP-1 has an important physiological role, apart from promoting embryonic development, as described below.

#### 7.1.1. Distinct Functions for the Two Different μ1 Subunits in *C. elegans*

The presence of two paralogous genes for the *C. elegans* μ1 subunits suggests the possible existence of two distinct AP-1 complexes. In fact, studies based on genetic mutants or RNAi knockdown experiments indicate the presence of two distinct AP-1 complexes whose functions may not be completely overlapping.

The characterization of Unc101, whose alleles have been previously identified as suppressors of a reduction-of-function mutation of *let-23* (an EGF receptor family tyrosine kinase in *C. elegans*) [52], shows that it encodes an ortholog of the AP50, mammalian μ1A [53]. The *unc-101* mutants are not embryonic lethal, but half of them die before reaching adulthood. Analysis of the larval stage of the *unc-101* mutants reveals that these mutants show pleiotropic phenotypes, including behavioral defects, such as uncoordinated movements and morphological abnormalities during vulval development [49,53].

In contrast, a knockdown of another μ1 subunit, apm-1, does not result in uncoordinated phenotypes, but shows developmental defects in the intestinal epithelial cells, suggesting a possibility of the distinct functions for unc-101 and apm-1, respectively. However, a simultaneous knockdown of both the μ1 subunits (or apm-1 knockdown in the *unc-101* background) leads to an embryonic lethal phenotype, demonstrating that unc-101 and apm-1 have a redundant role in embryonic development [49].

It remains to be seen whether the µ1 subunits carry out distinct functions similar to those of µ1A and µ1B subunits in mammals. Cell biological analyses demonstrate that Apm-1 and Unc-101 are expressed in the same cells during similar developmental stages. Knocking down the σ1 subunit results in the destabilization of both apm-1::GFP and unc-101::GFP at the protein level, suggesting that two distinct AP-1 complexes seem to exist in reality: AP-1^apm−1^ and AP-1^unc−101^. Functionally, a hybrid μ1 subunit, comprising unc-101 and the mouse μ1A, has been shown to rescue the phenotypes of *unc-101* [49]. Because the *apm-1* mutant shows strong phenotypes in the intestinal epithelial cells (see below), it is possible that the apm-1-containing AP-1 (similar to the AP-1B in mammals) plays a more important role in polarized sorting. The details of the specific molecular functions of these two μ1 subunits, however, remain elusive.

#### 7.1.2. The Role of AP-1 in Apical and Basolateral Polarized Sorting

During the larval stage, the *C. elegans* intestine generates a tubular structure by forming a lumen at the center. The lumen faces the apical membrane. In the apm-1 knockdown mutant, which arrests at the L1 larval stage, the basolateral membrane proteins, including SLCF-1 (a putative monocarboxylate transporter), are mislocalized [50,51]. Unexpectedly, however, a mistargeting of the apical membrane proteins, such as PEPT-1/OPT-2 (the oligopeptide transporter), AQP-4 (the water channel) and NHX-2 (the Na^+^/H^+^ exchanger), is also observed [50,51]. Furthermore, the apical molecules, including actin and PAR-6, and the apical lipids, including glycosphingolipids, are also mislocalized in the AP-1 mutants. These data demonstrate that AP-1 is indispensable for both apical, as well as basolateral sorting in the intestinal epithelium of *C. elegans*.

Mechanistically, it is pertinent to speculate that basolateral sorting is mediated through a direct recognition of the sorting signals by the μ1 subunit in *C. elegans.* although such a mechanism (similar to the one shown by the other μ subunits in mammals) has not been demonstrated directly. The mechanism by which AP-1 regulates apical sorting is unclear. A direct interaction of the apical sorting determinants with μ1, with a mechanism similar to that of basolateral sorting, seems unlikely [9,54]. Thus, AP-1 may be indirectly involved in apical sorting (e.g., by sorting and/or transporting proteins or molecules that control apical localization of proteins). Loss-of-function of AP-1 affects the sub-apical localization of rab11-positive endosomes that is thought to control the process of apical sorting. In fact, CDC42, an apical protein whose subcellular localization depends on rab11, was found to be mislocalized in the AP-1 mutants [51]. Therefore, a loss of AP-1 function could, in turn, perturb the TGN or endosomes controlling the apical targeting of proteins.

### 7.2. Fruit Fly (Drosophila melanogaster)

*Drosophila melanogaster* encodes every single AP-1 component. Thus, there is no epithelial-specific AP-1 (the counterpart of AP-1B in mammals) in *D. melanogaster*. However, the polarized sorting mediated by AP-1 has been reported to regulate the development of the fruit fly.

#### 7.2.1. AP-1 Controls Sensory Organ Development by Regulating the Basolateral Localization of *Sanpodo*

In *D. melanogaster*, the external sensory organ, which consists of four different types of cells, is developed through three rounds of cell divisions from a single cell type called the sensory organ precursor (SOP) cell [55,56,57]. During the first round, SOPs undergo asymmetric division to give rise to two daughter cells, *viz*. the pIIb (anterior) and the pIIa (posterior) cells. During this process, several cell fate determinants are segregated asymmetrically into two daughter cells, which results in the activation of distinct cell fate programs in each cell. One of the key molecules differentially regulated in these daughter cells is Notch, whose signaling is activated only in pIIa cells [57,58]. How is Notch signaling selectively activated in the pIIa cells, but not in the pIIb cells? This is in part due to the differential regulation of AP-1 in the pIIa and pIIb cells.

Benhra *et al.* carried out an RNAi screen to isolate new regulators of Notch signaling in the process of sensory organ development and identified AP-1 as a negative regulator of Notch signaling. The AP-1 loss-of-function mutation led to the misactivation of Notch signaling in pIIb cells (where this signaling is normally turned off), thereby demonstrating a *Notch* gain-of-function phenotype. Cell biological analysis revealed that the overall apical-basal polarity of the sensory organ cells was not disrupted in the AP-1 mutants. However, it was found that Sanpodo [59], an activator and a binding partner of Notch, which is normally localized at the endosomes and at the basolateral membranes in the pIIb and pIIa cells, respectively, is mislocalized and accumulated at the apical membrane of the pIIb and pIIa cells harboring the AP-1 mutants. Because Sanpodo binds to and activates Notch, the accumulation of Sanpodo-Notch in the apical plasma membrane results in the misactivation of Notch signaling in the pIIb cells in the absence of AP-1. Therefore, AP-1 controls the Notch-mediated cell fate specification through the regulation of basolateral sorting of Sanpodo in the sensory organ cells [60].

#### 7.2.2. Numb Regulates the AP-1-Mediated Sorting

What is the mechanism behind the AP-1-mediated Notch-Sanpodo regulation? Cotton *et al.* found that Numb, another key cell fate determinant known to inhibit Notch signaling [61,62,63,64], genetically and physically interacts with AP-1 [65]. Given that Numb is asymmetrically segregated only into the pIIb cells, where Notch signaling is inhibited [66], there may be a distinct regulation of AP-1 by Numb in the pIIb cells. In fact, Sanpodo is recycled back to the plasma membrane only in the Numb-negative pIIa cells, but not in the Numb-positive pIIb cells in wild-type, suggesting that Numb indeed prevents Sanpodo recycling [65]. In the absence of Numb, Sanpodo is recycled back to the plasma membrane in the pIIb cells, as well as in the pIIa cells [65]. These results indicate that Numb somehow inhibits the AP-1-mediated recycling of the Sanpodo-Notch complex at the endosomes in the pIIb cells, thereby preventing the activation of Notch signaling selectively in the pIIb cells. Because Numb is an adaptor molecule containing the PTB domain, the function of AP-1 can be modulated by the interaction with Numb. The detailed mechanism, however, remains unknown.

### 7.3. Zebrafish (Danio rerio)

The *D. rerio* genome encodes three μ1 subunits, *viz*. μ1A, μ1B and μ1C, respectively. Bioinformatics analysis reveals that *D. rerio* μ1A and μ1B show the highest homology to human μ1A and μ1B, respectively, whereas *D. rerio* μ1C is equally similar to human μ1A and μ1B, respectively [67]. RT-PCR demonstrates that μ1B is expressed in tissues containing polarized epithelium, such as the gut, pancreas, kidneys and testis, which is consistent with the distribution of mammalian μ1B. This tissue-specific expression is most pronounced during the developmental period. Interestingly, μ1B is also expressed in the liver, thereby suggesting a distinct function of μ1B in the zebrafish liver and/or a distinct function of the liver itself in zebrafish. In contrast, μ1A is not ubiquitously, but is specifically, expressed in tissues in which μ1B is not expressed (e.g., the brain, eye, skeletal muscles, heart and testis). The expression of μ1C is similar to that of μ1A [67,68].

#### 7.3.1. The Role of *D. rerio* AP-1B in the Development of Kidney and Liver Tissues Containing Epithelial Cells

Zebrafish injected with the μ1B morpholino, which possess kinky tails and exhibit a delayed development, show developmental defects in the kidney, gut and pancreas [68]. The μ1A/μ1B double-knockdown zebrafish demonstrate more severe defects in the kidney and also develop cardiac edema, which is probably due to defects in the kidney function [67]. These results suggest an essential role of *D. rerio* μ1B in the optimal development of kidneys. In contrast, the μ1B KO mice show no apparent defects in their kidneys (see below) [69]. Another difference between zebrafish and mice is the effect of the μ1B knockdown on the liver. Liver development is altered in *D. rerio* μ1B knockdowns [67,68], thereby suggesting a distinct function of the liver in mammals and fish, as discussed above.

#### 7.3.2. The Role of AP-1 in the Hair Sensory Epithelial Cells

Previous genetic screenings have identified mutants showing defects in auditory functions [70], and positional cloning has also identified apb1 (zebrafish β1 subunit) as the responsible gene [71]. The mutants show behavioral defects (e.g., circular swimming patterns and inability to maintain an upright resting position), suggesting that these mutations induce balance defects in addition to auditory deficits. Cell biological analyses revealed functional and morphological defects in the hair cells and sensory epithelial cells. These defects affect the auditory functions in the mutants. Furthermore, the Na^+^/K^+^-ATPase pump, a major Na^+^ regulator in these cells, is mislocalized to the apical membrane and also is reduced at the basolateral membrane, thereby resulting in the increase of intracellular Na^+^ [71]. Thus, AP-1 supports hair cell functions by controlling the basolateral sorting of the Na^+^/K^+^-ATPase in zebrafish.

It should be noted that in mammals, the Na^+^/K^+^-ATPase localizes at the basolateral membrane in hepatocytes [72] and kidney proximal tubular epithelial cells [73], but localizes at the apical membrane in RPE [74]. µ1B is not expressed in all three epithelial cell types. Furthermore, AP-1B has been shown to be dispensable for the basolateral localization of the Na^+^/K^+^-ATPase [32,75]. Thus, the role of AP-1 in controlling the localization of Na^+^/K^+^ ATPase is still less clear and could involve distinct tissue- and organism-specific mechanisms.

### 7.4. Mouse

As described earlier, there are two AP-1 complexes in mammals, *viz*. AP-1A and AP-1B. Both the AP-1 complexes share the γ, β1 and σ1 subunits. However, AP-1A harbors the ubiquitously expressed μ1A, whereas AP-1B harbors the epithelium-specific μ1B. Thus, AP-1B represents the epithelial cell-specific AP-1 complex, whereas AP-1A is expressed ubiquitously.

#### 7.4.1. The Essential Role of AP-1A in the Mouse Embryonic Development

Several knockout mice deficient in a subunit of AP-1 have been studied so far. Animals deficient in σ1B, one of the three σ subunits identified in mammals (σ1A-C), have been reported to grow normally without any severe developmental abnormalities, although impairments of some membrane trafficking processes, such as synaptic vesicle recycling, have been observed [76]. However, a recent study demonstrates that σ1B KO mice show lipodystrophy due to an impairment of adipogenesis [77]. A loss of the γ subunit, which is shared by both AP-1A, as well as AP-1B, causes a loss of the functional AP-1A/B complexes, resulting in an embryonic lethal phenotype at embryonic day 3.5 (E3.5), at which time, the maternal mRNAs and proteins start disappearing [43]. The μ1A KO mice, on the other hand, survive until E13.5 [44]. These results suggest that either form of AP-1 is required for cell viability and that AP-1A is essential for embryonic development, whereas AP-1B can replace its role until E13.5. In fact, exogenous µ1B expression in µ1A KO fibroblasts has been shown to partially restore a sorting defect in these cells [78]. However, as described below, AP-1B was found to be dispensable for embryonic development, but not for the normal functioning of epithelial cells in mice.

#### 7.4.2. AP-1B Regulates Polarity and Integrity of the Intestinal Epithelial Cells in Mice

The μ1B KO mice, which lack the AP-1B complex, develop normally until two weeks of age. However, after that, the KO mice start to show growth retardation, and 50% of the mice die within eight weeks. Cell biological and morphological examinations demonstrate a polarity defect in the intestinal epithelial cells of the KO mice, consistent with similar observations in the case of the *C. elegans* AP-1 mutants [50,51]. Furthermore, a mislocalization of the basolateral proteins, including EphB2, LDLR and E-cadherin, is evident, suggesting that AP-1B regulates the basolateral targeting of these proteins in mice, as expected from the observation in cultured epithelial cells [9,32,41,42]. The mislocalization of E-cadherin leads to the destabilization of the E-cadherin-β-catenin complex at the adherence junction. This, in turn, results in the translocation of free β-catenin into the nucleus, where it acts as a transcription factor together with TCF4 and upregulates the genes controlling cell proliferation. This leads to an excessive proliferation of the intestinal epithelial cells. This intestinal hyperplasia likely causes malfunction and subsequent malnutrition and ultimately causes growth retardation and possibly death. Thus, AP-1B controls the polarity and proliferation of the intestinal epithelial cells in mice [69].

Surprisingly, other tissues expressing μ1B, including the kidney (although its proximal tubules lack the expression of µ1B, as mentioned earlier [30]), are not apparently affected. The reason why the epithelial cells of the intestine become affected remains elusive. This could be due to a much higher proliferative property of the intestinal epithelial cells [79], which may require a tighter regulation by AP-1B. An alternative, but not a mutually exclusive, possibility is that the other AP complexes, such as AP-1A [38] or AP-4 [80], as well as other molecules or machineries, could compensate for the loss of AP-1B, reflecting a surprising plasticity, as well as a small amount of redundancy in polarity control in the epithelial cells and tissues, respectively.

#### 7.4.3. Ectopic Apical Formation in AP-1-Deficient Animals

In addition to the mis-sorting of the basolateral proteins, the mislocalization of the apical proteins, including sucrase or villin, to the lateral plasma membrane was unexpectedly observed in the μ1B KO intestinal epithelial cells. This was accompanied with the appearance of an ectopic microvilli-like structure in the basolateral domain, as well as in the subapical cytoplasm [69]. This “lateral to apical conversion” was also seen in the *C. elegans* AP-1 mutants [50,51], suggesting that AP-1B (or AP-1 in *C. elegans*) is required for the localization of some of the apical proteins. As discussed earlier, however, it is not clear whether AP-1B directly controls the apical protein sorting and transport. Given that similar, but not equal, ectopic apical formation phenotypes were observed during the loss of function of other proteins, including rab8 [81], clathrin [50], PAR-6 [82] and enzymes involved in the biosynthesis of glycosphingolipid synthesis [83], it seems that AP-1B may not be a direct regulator of apical sorting. Rather, AP-1B may affect apical targeting indirectly through abnormal basolateral targeting. AP-1B could presumably act as an upstream regulator for the apical sorting regulatory machinery.

### 7.5. Human

To date, there is no direct evidence that mutations in AP-1B cause any diseases in humans. However, two studies implicate a possible involvement of AP-1B in human diseases [84,85].

#### 7.5.1. Crohn’s Disease

In addition to the basolateral proteins described above, cytokine receptors, such as the interleukin 6 signal transducer (IL-6st) and the poly-immunoglobulin receptor (pIgR), get mislocalized in the colonic epithelial cells of μ1B KO mice. These changes likely caused immune dysfunctions; compromised cytokine responses, a reduction of the antimicrobial peptide expression and an impairment of immunoglobulin A transcytosis. As a result, the barrier function of the colon is compromised, leading to the enhanced translocation of bacteria into the mucosa, which causes chronic inflammation in the μ1B KO colon. This inflammatory phenotype is similar to the one observed in Crohn’s disease, a form of inflammatory bowel disease. Interestingly, the expression level of μ1B mRNA is reduced in Crohn’s disease patients [84]. These data indicate that AP-1B secures gut immune homeostasis and also suggest that AP-1B could be involved in the pathogenesis of Crohn’s disease.

#### 7.5.2. Colorectal Cancer

The reduction of epithelial cell polarity is often associated with tumorigenesis [85]. Given that AP-1B controls the polarity and proliferation of intestinal epithelial cells in mice, intestinal tumorigenesis might involve AP-1B. In fact, a lower expression of μ1Β was reported in human colorectal cancer tissues. Furthermore, such a reduction of the μ1B expression correlated with the nuclear localization of β-catenin [86]. These observations are consistent with the phenotype observed in the μ1B KO mice; intestinal hyperplasia and the loss of cell polarity, concomitant with the enhanced nuclear translocation of β-catenin [69]. Thus, the AP-1B-mediated regulation of cell polarity and proliferation could help prevent tumorigenesis.

## 8. Concluding Remarks

Since its discovery in 1999, AP-1B has been established as one of the central players in the polarized sorting of epithelial cells. In addition, much progress has been made, including a recent discovery of the unexpected role of AP-1A in polarized sorting, which brought more questions than answers. How does an epithelial cell distinctly employ AP-1A and AP-1B? What is the mechanistic difference? Why do some epithelial cells evolve to have μ1B?

Studies using animal models clearly highlight the importance of AP-1B or AP-1 (in *C. elegans* and *D. melanogaster*) in the *in vivo* regulation of polarized sorting. These studies have brought us novel insights into the physiological roles of AP-1B (including developmental differentiation and gut immune homeostasis) and have highlighted some unexpected functions, such as the regulation of apical sorting. However, we still do not fully understand the differential regulation and expression of AP-1A and AP-1B during development. Additionally, the mechanism by which the lack of µ1B expression in some epithelial cells, such as hepatocytes, kidney proximal tubules and RPE, contributes to the normal physiology in the respective tissues (e.g., the liver, kidneys and the retina) also remains elusive. More importantly, the analyses of the μ1B KO mice has revealed that the functional impairment of the AP-1B-mediated sorting might be involved in the pathogenesis of human diseases, such as Crohn’s disease or colorectal cancer. Further studies with the above-mentioned animal models, in combination with the cell culture systems, could help us understand the role of AP-1B. This would shed some light on the various kinds of pathological mechanisms and processes, thereby facilitating the process of drug discovery.
